# Meta-scale mountain grassland observatories uncover commonalities as well as specific interactions among plant and non-rhizosphere soil bacterial communities

**DOI:** 10.1038/s41598-018-24253-x

**Published:** 2018-04-10

**Authors:** Erika Yashiro, Eric Pinto-Figueroa, Aline Buri, Jorge E. Spangenberg, Thierry Adatte, Helene Niculita-Hirzel, Antoine Guisan, Jan Roelof van der Meer

**Affiliations:** 1 Department of Fundamental Microbiology, University of Lausanne, 1015 Lausanne, Switzerland; 2 Department of Ecology and Evolution, University of Lausanne, 1015 Lausanne, Switzerland; 3 Institute of Earth Surface Dynamics, University of Lausanne, 1015 Lausanne, Switzerland; 4 Institute of Earth Sciences, University of Lausanne, 1015 Lausanne, Switzerland; 5 Institute for Work and Health, University of Lausanne and Geneva, 1066 Epalinges Lausanne, Switzerland

## Abstract

Interactions between plants and bacteria in the non-rhizosphere soil are rarely assessed, because they are less direct and easily masked by confounding environmental factors. By studying plant vegetation alliances and soil bacterial community co-patterning in grassland soils in 100 sites across a heterogeneous mountain landscape in the western Swiss Alps, we obtained sufficient statistical power to disentangle common co-occurrences and weaker specific interactions. Plant alliances and soil bacterial communities tended to be synchronized in community turnover across the landscape, largely driven by common underlying environmental factors, such as soil pH or elevation. Certain alliances occurring in distinct, local, environmental conditions were characterized by co-occurring specialist plant and bacterial species, such as the *Nardus stricta* and Thermogemmatisporaceae. In contrast, some generalist taxa, like *Anthoxanthum odoratum* and 19 Acidobacteria species, spanned across multiple vegetation alliances. Meta-scale analyses of soil bacterial community composition and vegetation surveys, complemented with local edaphic measurements, can thus prove useful to identify the various types of plant-bacteria interactions and the environments in which they occur.

## Introduction

Plant and soil bacterial communities are intimately related, with notable implications for ecosystem productivity, functioning and global change^1–4^. Soil bacteria and fungi are key players in nutrient cycling^5,6^; they further decompose and transform organic matter to compounds more readily taken up by plant roots and other soil organisms^2,7^. The available soil nutrients allow plants to grow and flourish and, in turn, plants help to sequester atmospheric carbon, produce oxygen, and stabilize the soil to prevent excessive erosion^3,8^. Plant-microbe interactions can drive changes in biogeochemistry that strongly influence cycling of carbon and major nutrients^7,9^, and, as such, have important implications for ecosystem function at broad scales including productivity and climate feedback^10,11^.

Plant-bacteria interactions have been extensively studied in agricultural crops and model plants such as *Arabidopsis thaliana* and *Medicago truncatula*^12^. Plant root exudates promote the proliferation of specific bacteria and fungi leading to rhizosphere-consortia that may be favorable for plant growth^13^. Much research so far has described plant-microbe interactions in the rhizosphere. Such interactions can either occur directly through symbiosis (e.g., nutrient delivery, phytohormones), or indirectly via changes in soil biogeochemistry^14,15^. Rhizosphere bacterial communities are often specific to the respective plant species, suggesting ecologically well-established relationships^16–19^. They are distinct from the bacterial communities found in the surrounding soil^20–22^, but form reproducibly from such communities as plant roots develop^23,24^.

While research on the rhizosphere has demonstrated plants influencing and selecting local microbial communities, the effect of plants on microbes has also been noted on a wider scale and outside the rhizosphere^25–28^. The relationship between plant and soil bacterial diversity outside the strict rhizosphere may arise from both direct (e.g., root exudates, phytohormones) or indirect effects such as litter input^26^. Since soil types differ across the landscape, one would expect that plants at different sites enrich their root communities from whatever bulk soil community is available locally, possibly favoring specific plant-bacteria interactions as a function of site characteristics and the native resident microbial community. While difficult to distinguish at a single site, across many sites such plant-soil bacterial correlations may be more clearly discerned. Studying plant-soil bacteria co-occurrences at the landscape scale is thus a reasonable first step to uncover the types of favorable or unfavorable interactions. One could, for example, focus on specific highly abundant plant species that co-occur with particularly abundant bacterial groups, or compare plant vegetation types with bacterial community “types” at a “meta” level.

Since both plants and bacteria are influenced by environmental factors, it is difficult to tease apart co-occurrences that are the result of biotic interactions from those resulting from similar influence of environmental factors^29^. Arguably, at least the same environmental determinants would yield generally similar plant bacterial co-patterning, for example, both showing particular pH dependencies^30,31^. However, co-patterning of plant species and bacterial groups across multiple sites can also point to more specific interactions^32,33^. Mountain ecosystems can be very useful observatories to study plant-bacterial associations and putative interactions at the landscape scale, as they are characterized by steep environmental gradients and rugged topography, creating numerous microclimatic habitats with high plant and bacterial community diversity, changing across short distances^34–36^. Using joint observations of plants and bacteria at a series of locations across a mountain landscape, one can test for *general* co-patterning between plant and bacterial communities, as well as identify individual plant species and bacterial groups exhibiting more *specific* co-occurrences.

Recent studies have demonstrated that relatively distinct vegetation habitats or vegetation alliances can harbor significantly different belowground soil bacterial communities^37,38^. Furthermore, while several studies have investigated the drivers of microbial biogeography across different ecosystems^31^ or among similar vegetation types^25^, no study has attempted to explore plant-bacteria interactions from a large set of measurements of soil bacterial communities with respective aboveground plant cover across a wide range of vegetation types. We took advantage of the precise description of 15 different vegetation alliances, using exhaustive inventories of plant species in plots across a well-studied area in the Western Swiss Alps^30,39^, as well as of a recent characterization of the bacterial communities and edaphic factors in the bulk top soil of a large number of sites, spatially and randomly distributed within successive elevation and environmental strata across the area^36^. In the present study, we compared the bacterial community data from 100 sites to their plant species distributions and vegetation alliances, and further to local soil environmental factors^36^. First, we questioned whether the structures of the bacterial communities at the sites– exemplified by diversity of operational taxonomic units (OTUs), co-vary with the prevailing vegetation types (here, at the level of *alliances*). Secondly, we examined correlations between cover abundance of specific plant species and bacterial OTU relative abundances across all sites, in order to infer potential generic and specific plant-bacteria interactions. These were then further analyzed and corrected for the extent of variation explained by shared environmental preferences.

## Results

### Geographic co-variation of bacterial communities and vegetation alliances

**Figure 1:**
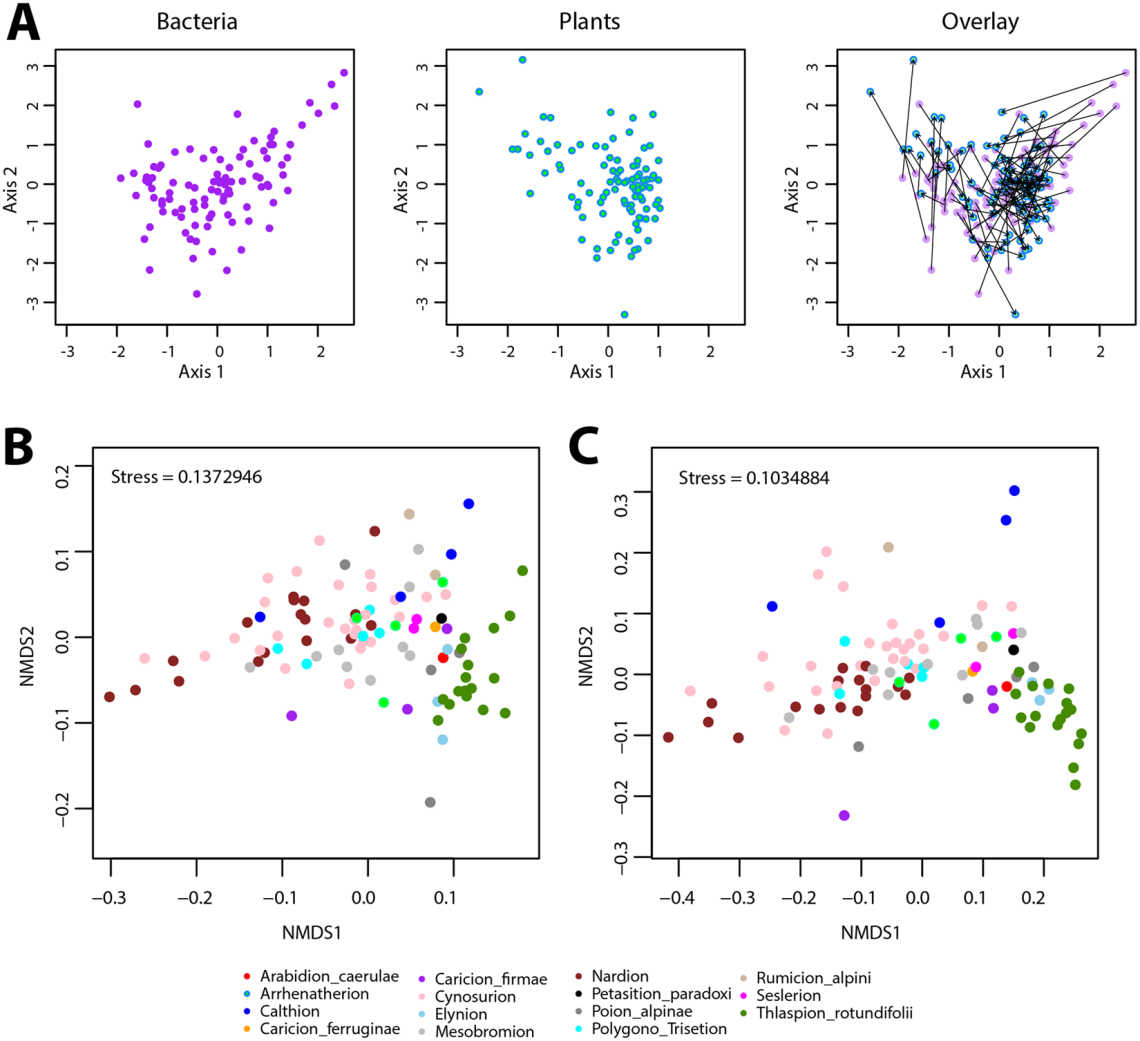
Co-patterning of mountain grassland plant and bacterial communities. (**A**) Coinertia analysis based on the plant cover-abundance NMDS and bacterial community structure NMDS scores. The bacterial (purple) and plant (blue) components of the coinertia analysis are shown separately and overlaid across the ordination space for easier visualization, with arrows connecting bacteria and plant dots from the same site. (**B**) and (**C**) Co-patterning of bacterial communities per vegetation alliance, plotted in NMDS based on bacterial community structure and (**C**) on community membership. Dots (representing each site) are color-coded according to their prevailing vegetation alliance, but vegetation alliance information was not used for the NMDS calculation.

We examined global co-variation of non-rhizosphere grassland soil bacterial communities (from their OTU diversity) and aboveground plant cover, across 100 sites in the Western Swiss Alps region. The sites had been characterized previously in terms of 56 edaphic factors and bulk soil bacterial community composition^36^. Co-inertia analysis indicated congruence of bacterial community composition and plant cover abundance in non-metric multidimensional scaling (NMDS) ordination space for most sampling sites, with certain exceptions (Fig. 1A). Notably, groups of similar bacterial community composition corresponded to groups of similar plant community composition (Fig. 1A, overlay, note the arrows connecting bacterial and plant communities at the same site). This may be the result from preferences of bacterial and plant communities for similar environmental and climatic factors, or from biotic dependences (see below).

A clearer picture of bacterial-plant co-patterning was obtained when vegetation plots were classified at the level of alliances. Differences in the bacterial community composition across all sites plotted in NMDS ordination space and overlaid by coloring of vegetation alliances showed clear co-variation (e.g., Fig. 1B, green dots – Thlaspion rotundifolii), as well as some overlaps (Fig. 1B, e.g., Cynosurion and Nardion). The areas covered by the points corresponding to each alliance in the ordination space (calculated by *ordihulls*) was significantly smaller than the total area covered by all bacterial community samples (*P* < 0.0001 for the NMDS ordihulls of both the weighted and unweighted UniFrac matrices). This suggests that, within the ordination space defined by axes 1 and 2, the bacterial communities within the respective vegetation alliances are significantly more similar to each other than between different alliances. The co-patterning of plant alliances and bacterial communities in ordination space likely results from factors other than chance alone, such as underlying environmental factors and/or positive or negative interactions between species in the two groups. The NMDS plots from both the weighted and the unweighted UniFrac matrices displayed similar co-localized trends between bacterial communities and vegetation alliances (Fig. 1B,C), indicating that at most sites, both bacterial OTU relative abundances (Fig. 1B) and the bacterial community membership (Fig. 1C) co-vary with the vegetation alliances.

### Effects of underlying environmental factors

**Figure 2:**
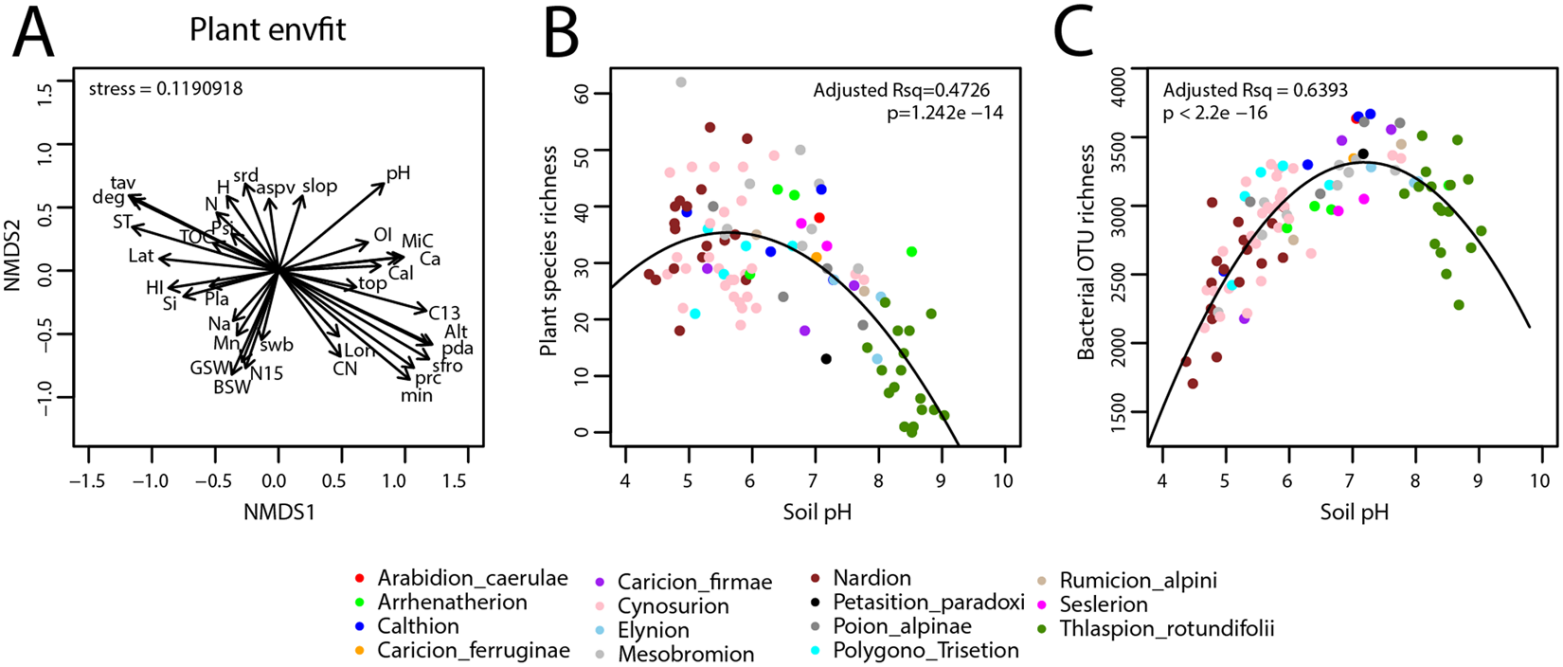
Effect of abiotic factors on the plant and bacterial communities. (**A**) General effect of abiotic factors on the plant community structure, determined from the cover-abundance at each site and the environmental variables within NMDS ordination space using *envfit()*, and using previously published data^30,36,40^. Arrows indicate relative degree and directionality of effect. ST, soil temperature at depth −5 cm; pH, soil pH; C13, stable isotopic carbon ratio (mL^−1^ vs VPDB); N15, stable isotopic nitrogen ratio (mL^−1^ vs N_2_ in air); H, bulk hydrogen content (wt %); N, bulk nitrogen content (wt %); CN, C:N ratio; HI, hydrogen index (mg HC g^−1^ TOC); OI, oxygen index (mg CO_2_ g^−1^ TOC); TOC, total organic carbon content (wt %); MiC, mineral carbon content (wt %); BSW, bulk soil gravimetric water content (40°, %); GSW, sieved soil gravimetric water content (105 °C, %); Si, SiO_2_ (wt %); Mn, MnO content (wt %); Ca, CaO content (wt %); Na, Na_2_ content (wt %); PSi, phyllosilicates (%); Pla, plagioclase-Na (%); Cal, calcite (%); Lon, longitude (x, Swiss coordinate system); Lat, latitude (y); Alt, elevation (m); slop, terrain slope (°); aspv, sine transformed direction that a slope faces; top, topographic position; deg, annual degree days (day × deg); min, monthly moisture index (0.1 mm month^−1^); srd, daily average of global potential shortwave radiation per month (kJ day^−1^); tav, monthly average temperature (°C × 100); swb, annual average site water balance accounting for soil properaties (0.1 mm year^−1^); sfro, annual average number of frost days during the growing season (day × 100); pda, number of precipitation days per growing season (day); prc, monthly mean precipitation sum (mm). (**B**) Plant species richness and (**C**) bacterial OTU richness at each site plotted against soil pH. The dots are color-coded *a posteriori* according to the vegetation alliance to which the sites belong. Lines in panels B and C display quadratic trend-lines with their adjusted *R*^*2*^ (Rsq) and *P-*values.

In order to dissect possible underlying influences from environmental factors on both vegetation and bacterial soil community diversity, we examined the effects of 56 environmental parameters, previously determined at the same sites^36,40^, on both plant and bacterial community structures across the mountain landscape. Taken individually, plant community structure was most strongly influenced by elevation (Fig. 2A, “Alt”) and environmental factors related to elevation (e.g., Fig. 2A, average number of frost days *sfro*, monthly moisture index *min*), but less by soil pH (Fig. 2A, but see Buri *et al*.)^40^. This is evident from the vector length in NMDS space, with envfit R^2^ values of 0.6034 for elevation, and 0.3876 for soil pH. In contrast, and as previously demonstrated, the soil bacterial community diversity across the sites was more dependent on soil pH and less on elevation^36^. It is worth noting that the 56 environmental parameters cross-correlate to varying extents, resulting in 18 correlation clusters^36^. For instance, pH notably correlates to CaO and calcite content, bulk C:N ratio and organic matter content, whereas elevation correlates to soil temperature and annual number of frost days^36^.

**Figure 3:**
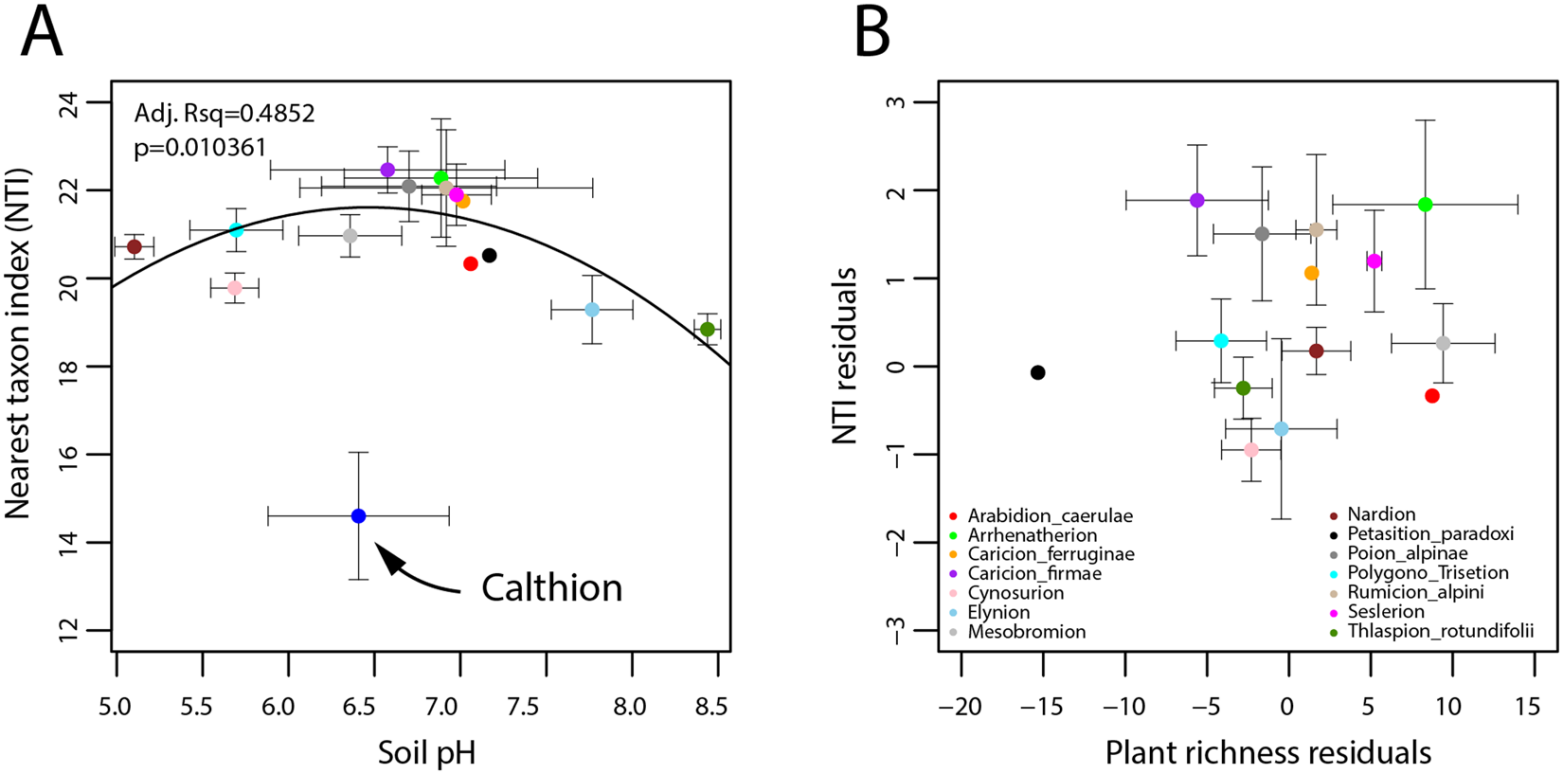
Effect of the environment on the evolutionary history of mountain grassland soil bacterial communities. (**A**) Mean bacterial nearest taxon index (NTI) values across vegetation alliances versus soil pH. (**B**) Bacterial NTI residuals versus plant species richness residuals after removing the pH effect on both, and excluding the Calthion alliance outlier. Dots are color-coded by alliance, whereas error bars represent the standard error within each vegetation type. The line in panel A displays the quadratic trendline across all the mean NTI values, excluding the Calthion samples, with adjusted *R*^*2*^ (Adj. Rsq.) and *P-*value as indicated.

Plant species richness across the sites displayed a quadratic trend as a function of the soil pH, similarly to bacteria, but the pH at which highest plant species richness occurred was lower (pH 5.5–6.0) than for bacteria OTU richness (pH 7.0–7.5) across the same sites (Fig. 2B,C). Grouping the bacterial community’s Nearest Taxon Index (NTI) per alliance showed that all of the alpine grassland soil bacterial communities were highly phylogenetically clustered and not overdispersed (Fig. 3A). However, the sites with harsher environmental conditions resulted in relatively more overdispersed bacterial communities, notably those sites that were either water-logged and with anoxic soil conditions (Fig. 3A, Calthion vegetation alliance) or high elevation, highly exposed, and poorly vegetated (Thlaspion rotundifolii, Elynion, and Petasition paradoxi vegetation alliances). When excluding the sites with a Calthion vegetation alliance, the alpine grassland soil bacterial community NTI values followed a quadratic trend as a function of soil pH (Fig. 3A), similarly to what had been observed using other diversity indices such as OTU richness and Faith’s phylogenetic diversity (Figs 2C and S1). Correlations between the bacterial NTI and plant species richness were much less profound when the pH effect was removed, which can be seen from the residuals from the quadratic linear fits (Figs 3B and S2). However, even after removing the effect of pH, the bacterial communities at the vegetation alliances whose mean soil pH was near-neutral were significantly more clustered than those from alliances whose mean soil pH was non-neutral (Kruskal-Wallis test, *P* = 1.67 × 10^−5^).

### Specific bacterial indicators of vegetation alliances

**Figure 4:**
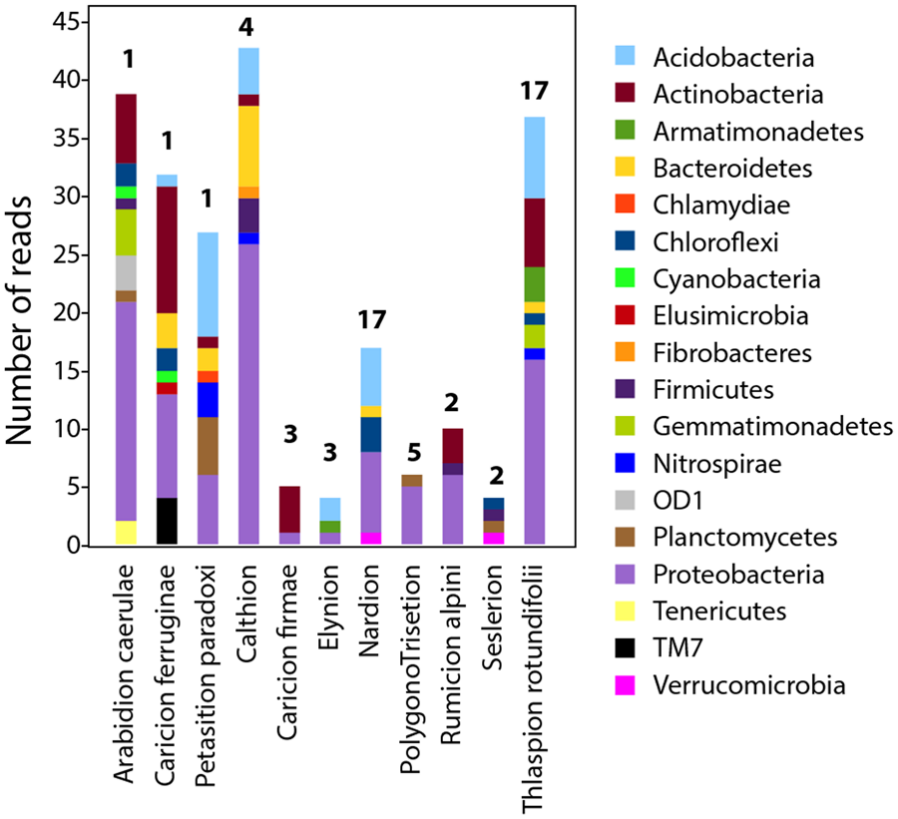
Taxonomic profile of bacterial indicator OTUs across vegetation alliances. The values above the bars indicate the number of sites belonging to that respective alliance. Note that alliances with only 1 site are not taken into consideration for alliance correlations.

Bacterial indicator OTUs could be identified from eight out of the 12 vegetation alliances which contained more than one site in the study area (Table 1, Figs S3 and S4). The indicator OTUs identified for three other vegetation alliances were likely site-specific individuals because these alliances each included only one site. For the vegetation alliances occurring multiple times, the indicator OTUs were predominated by members of the Proteobacteria (Fig. 4, purple stacks). Acidobacteria, Actinobacteria and Bacteroidetes were also highly present. In general, a relatively large diversity of taxa was represented. The largest number of indicator OTUs was associated with the Calthion and Thlaspion rotundifolii alliances, followed by the sites with the Nardion and Rumicion alpine alliances. The alliances Calthion, Elynion, Nardion, Polygono trisetion, Rumicion alpini, Seslerion, and Thlaspion rotundifolii were distinct enough that indicator organisms could be identified for both plant and bacterial groups (Tables 1 and S1).

**Table 1:** Bacterial and plant indicators across the Alpine grassland vegetation alliances.

Alliance^a^	Habitat category	Habitat subcategory	No of Sites	No of indicator bacterial OTUs	No of indicator plant species
Arabidion caerulae	Grasslands and prairies	Snowy combe of limestone	1	39	5
Caricion ferruginae	Grasslands and prairies	Unimproved calcareous grassland	1	32	8
Petasition paradoxi	Glaciers, rocks, screes and moraines	Wet limestone scree	1	27	4
Arrhenatherion	Grasslands and prairies	Low altitude hayfield	4	0	1
Cynosurion	Grasslands and prairies	Low and mid-altitude pastureland	27	0	0
Mesobromion	Grasslands and prairies	Semi-dry medio-European grassland	9	0	2
Poion alpinae	Grasslands and prairies	Alpine and subalpine fertile pastureland	4	0	2
Calthion	Shores and humid zones	Buttercup prairie	4	43	6
Caricion firmae	Grasslands and prairies	Dry limestone lawn of firm sedges	3	5	0
Elynion	Grasslands and prairies	Lawn on windy ridges	3	4	1
Nardion	Grasslands and prairies	Sparse and acidic pastureland	17	17	1
Polygono Trisetion	Grasslands and prairies	Mountain hayfield	5	6	1
Rumicion alpini	Pioneer vegetation in anthropogenically disturbed areas	Alpine and subalpine cattle resting areas	2	10	1
Seslerion	Grasslands and prairies	Dry limestone lawn of seslerie	2	4	3
Thlaspion rotundifolii	Glaciers, rocks, screes and moraines	High altitude calcareous scree	17	37	2

^a^Alliance information according to Delarze & Gonseth (2008).

**Figure 5:**
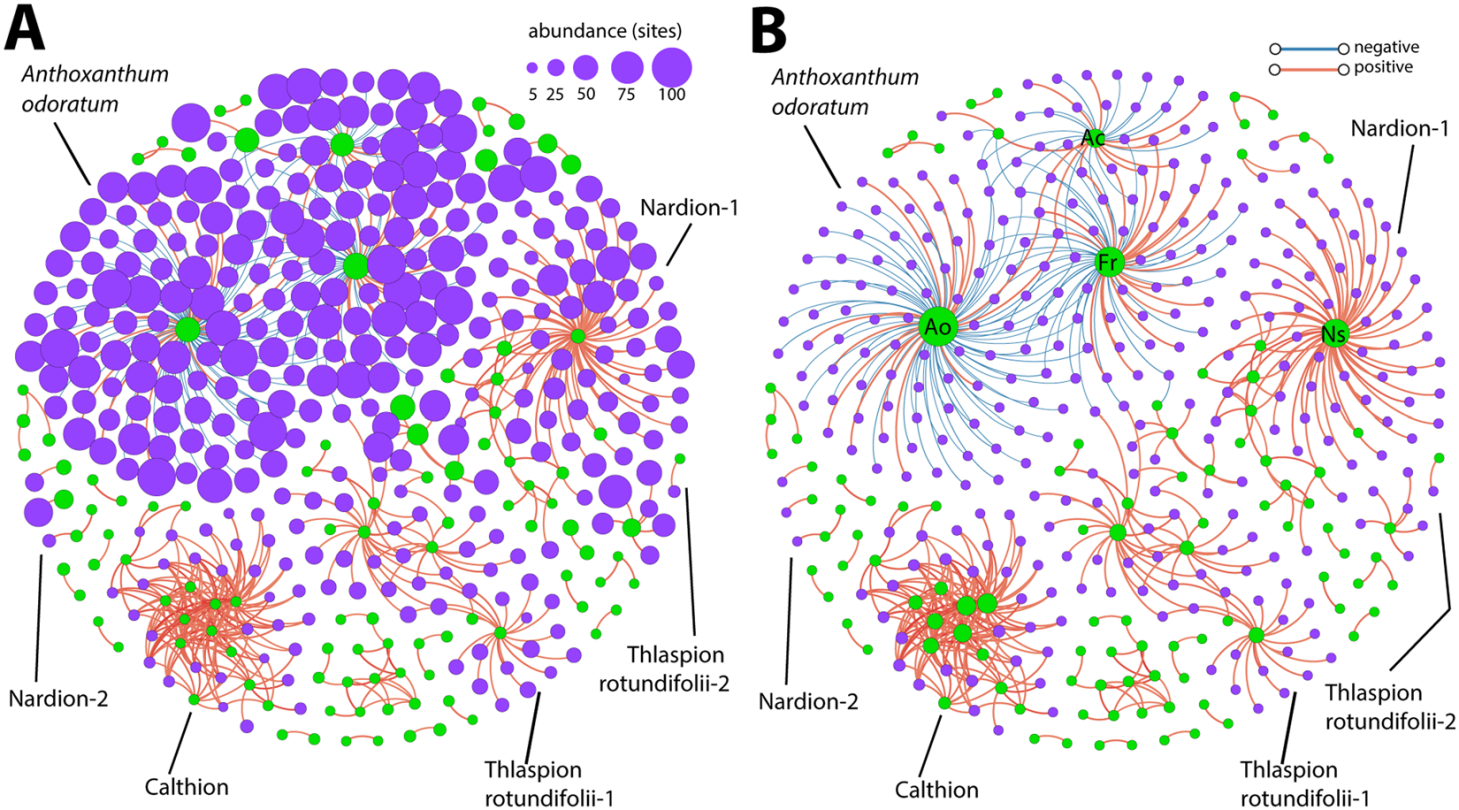
Correlation network analysis of the interactions among plant species alone and with bacterial OTUs, with node sizes in (**A**) indicating the number of sites where the plant species or bacterial OTU occurs, and (**B**) the degree of connectivity (the number of edges). Plant species nodes in green, bacterial OTUs nodes in purple. Edges represent the FDR-corrected positive (red) or negative (blue) Spearman’s correlations between plant species and bacterial OTUs, or among plant species. Only interactions with Spearman correlations of ≥0.6 or ≤−0.6 are indicated. Striking recognizable clusters are named according to abundant plant names, or vegetation alliances. Ao, *Anthoxanthum odoratum* aggr., Fr, *Festuca rubra* aggr., Ac, *Agrostis capillaris*, and Ns, *Nardus stricta*. Lines point to the most important alliances and clusters mentioned in the main text.

Among those bacterial OTUs whose relative abundances were directly correlated with plant species cover-abundance, the more frequently occurring bacterial OTUs were generally associated in clusters with the more frequently occurring plant species, and the less frequently occurring OTUs with less prevalent plant species (Fig. 5A, node sizes). Notably, however, bacterial OTU associations with the plant species *Anthoxanthum odoratum* aggr. (*Ao*) were dominated by negative correlations (Fig. 5B, blue edges), whereas those with *Festuca rubra* aggr. (*Fr*) *Agrostis capillaris* (*Ac*), *Alchemilla vulgaris aggr*. and *Trifolium pratense L*. contained both positive (Fig. 5B, red edges) and negative correlations. Interactions with all the other plant species were dominated by positive correlations (Figs 5B and S5). Generally, the most frequently occurring plant species in the area also had the highest number of correlations (positive and negative) with bacterial OTUs (Figs 5B and S5). However, in one cluster representative of the Calthion alliance, many plant species correlated with each other and inter-correlated with the same set of bacterial OTUs (Figs 5 and S5, Table S1 and Dataset 1). The bacterial OTUs and plant species in this cluster also occurred in relatively few sites (3–6 per species; Fig. 5A). Bacterial OTUs associated with this cluster included various anaerobic and putatively methanotrophic taxa (Bacteroidales, Chromatiales, Desulfobacterales, Entotheonellales, Methylococcales, Rhodocyclales, Solibacterales, Syntrophobacterales, and Fibrobacteres TG3), as well as three Nitrospirales OTUs.

Characteristic plant species of the Nardion and Thlaspion rotundifolii alliances as determined by Delarze & Gonseth^39^, and indicator bacterial OTUs present in the corresponding soils could be identified among different clusters in the correlation network (Figs 5, S6, Table S1 and Dataset 1). Interestingly, there was a large presence of the little known bacterial family Thermogemmatisporaceae in the clusters associated with the Nardion alliance. The other plant-bacterial clusters in the correlation network consisted of plant species that were either widely distributed across multiple vegetation alliances and were not characteristic of a single habitat, and/or the bacterial indicator OTUs were not present or representative of multiple vegetation alliances (Dataset 1). Some of the identified plant-only clusters belonged to specific alliances based on the presence of indicator plant species (Table S1 and Fig. S6).

### Environmental factors underlying specific bacterial OTU-plant species co-patterning

**Figure 6:**
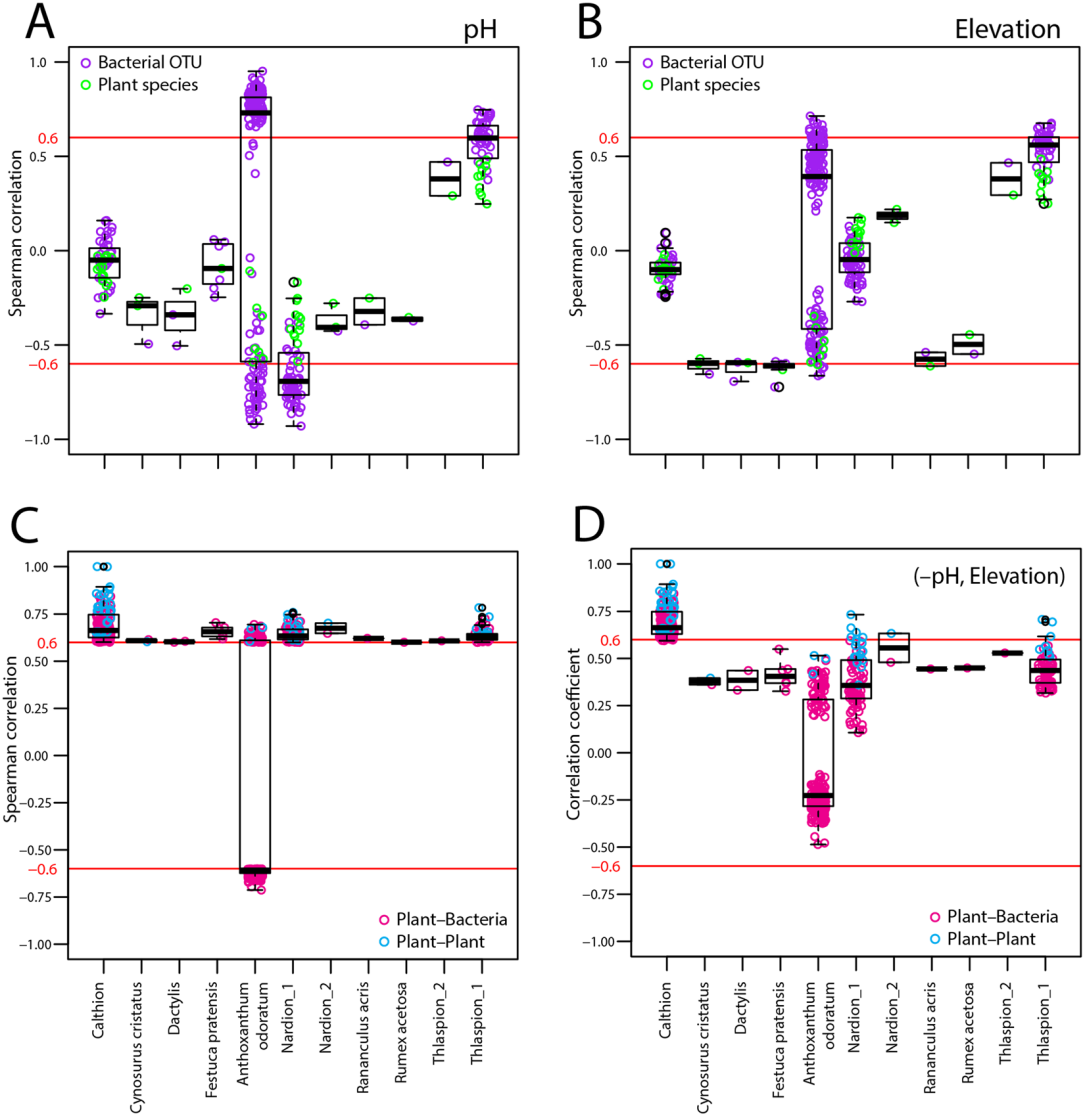
Dependency of bacterial OTU and plant species correlations within the respective clusters, as defined in Fig. 5, on underlying environmental variables. (**A**,**B**) Spearman correlations between individual plant species (green circles) or bacterial OTUs (purple) with soil pH or elevation, respectively. (**C**) Spearman correlations of plant-bacteria (magenta) and plant-plant (cyan) associations. (**D**) Partial Spearman correlations of (**C**) after removing the effect of soil pH and elevation. Plots of individual correlations (circles) are overlaid by box plots showing the 25%− and 75%− percentile, median, and outliers. Correlation cluster names for all panels indicated on the x-axis of (**C**,**D**). Red lines point to the thresholds, above or below which correlations are depicted in Fig. 5.

Environmental factors also displayed relatively strong correlations with plant species and bacterial OTUs in the correlation network analysis (Figs 6 and S7). Notably, soil pH correlated strongly with many bacterial OTUs in the *Anthoxanthum odoratum* cluster (Fig. 5), while the other plant species in this cluster displayed fairly strong correlations with both soil pH and elevation (Fig. 6A,B). In the acidic sites of the Nardion alliance, soil pH was a strong negative driver of both plants and bacteria (Fig. 6A). The organisms in the high-elevation, alkaline sites of the Thlaspion rotundifolii alliance also displayed relatively high correlations with both soil pH and elevation (Fig. 6A,B), as well as with the soil hydrogen index, nitrogen content, soluble phosphorus content, total organic carbon content, and δ^13^C-values (Fig. S7, the elevation dependence of δ^13^C was discussed in ref ^36^). It is worth noting that soil hydrogen index, nitrogen content, soluble phosphorus content, and total organic carbon content cross-correlate across the area, but do not correlate with soil pH or elevation^36^. Bacterial OTU and plant species within the four other clusters of Fig. 5 were as strongly correlated with elevation as they were among their respective members (Fig. 6B,C).

Removing the effect of the two most prominent drivers, soil pH and elevation, by partial correlation analysis confirmed that, for the Calthion alliance, the plant-bacteria associations are quite specific and unaffected by the removal of pH and elevation (Fig. 6C,D). The remaining Spearman correlations after partialing out pH and elevation in the other clusters of Fig. 5 were much weaker, but still clearly showed on average the positive associations between plants and bacteria or between plants (Fig. 6D). Other environmental variables (bulk soil water and total organic carbon content, hydrogen index, δ^13^C, mineral carbon, total N and soluble P) were less important for determining the Spearman correlations (Fig. S7), and are not dependent on soil pH or elevation^36^.

## Discussion

In this study we identified both commonalities and specific interactions between bacterial and plant communities across 100 sites and 15 distinct grassland plant alliances in the western Swiss Alps. We inferred possible interactions between bacterial communities and vegetation alliances, and between more specific members of each group, while removing possible underlying effects of commonly acting environmental factors. Our results showed that plant and bacterial communities tend to synchronize across the landscape, and illustrate how environmental gradients differentially influence the ecology of both organism groups. Our results support the idea that specific plant-bacteria interactions may become favored through enrichment from the bulk soil as a function of site characteristics and the native resident microbial community.

Plant-bacteria interactions have been studied before at the landscape scale^25–28^, but our work uniquely spanned an entire mountain region with wide environmental gradients. It further highlights the usefulness of phytosociologically assigned vegetation alliance information as a proxy for plant community composition for the analysis of the biogeography of belowground bacterial communities. We showed that plant vegetation alliances across a wide elevation gradient, characterized by large gradients in other topo-climatic and physico-chemical parameters^34,36^, clustered within the bacterial community ordination space. This strongly suggests that the bacterial and plant communities share similar spatial and environmental distributions across the landscape, with both bacterial membership and relative abundances often highly specific to vegetation types (Fig. 1B,C). The advantage of using vegetation alliances instead of only single plant species is that they provide categorical information about the overall plant community composition at any given site, and take the community as a whole into account^39^. The observed trend is highly consistent across as many as 15 different vegetation alliances within a whole region (with an area of ca. 700 km^2^), despite finding mixed situations where vegetation alliances overlap among soil bacterial community diversity (Fig. S3). This may be the reason for lack of indicator taxa in mixed situations corresponding to a transition between two vegetation alliances^39^. Correlations of bacterial communities and vegetation types had been detected before on smaller datasets, for example by Chu *et al*.^37^ in birch hummock, dry heath, and wet sedge vegetation types, and by Geremia *et al*.^38^ in grasslands between two vegetation habitats across the Carpathians and the Alps, but never before across such a wide variety of alliances as reported here.

In 8 out of 12 vegetation alliances that occurred in more than one instance within our study, the bacterial community structure in the alpine grassland soil was specific enough to their plant alliances for indicator OTUs to be identified. These plant alliances with significant bacterial indicators were characterized by acidic soil conditions, high elevation exposed environments, very humid and water-logged areas, and relatively dry grasslands and pasturelands^36,39^. On the contrary, those alliances where indicator OTUs could not be found were generally low to high elevation pasturelands and hayfields with less distinct characteristics^39^, and where alliances tended to overlap in bacterial community ordination space (Fig. 1B,C).

Mountain grassland plant species with the strongest associations to bacterial OTUs belonged to two main categories: widely distributed generalist plants present among multiple vegetation alliances, and specialist plant species that were mainly restricted to specific vegetation alliances, notably those of the Nardion, Thlaspion rotundifolii, Calthion, and tentatively Cynosurion alliances. This suggests that certain vegetation alliances may have evolved highly specific, and potentially beneficial, plant-bacterial interactions due to the more extreme environmental conditions that require cooperation rather than competition. The possibly increasing importance of plant-microbe interactions in stressful environments may be an aspect worthwhile to study further. In this respect, the grasslands characteristic of the Nardion alliance experience a large degree of cattle trampling, while the high elevation sites with Thlaspion rotundifolii alliance persist on unstable substrates and cold conditions, and the sites with Calthion alliance are frequently inundated^39^.

Plant species cover abundance and bacterial OTU relative abundances were highly co-varying across sites with the Calthion alliance. This suggests that the life history strategy of these organisms is co-evolving and highly dependent on specific environmental conditions, likely anoxic soil, given the characteristic anaerobes and putative methanotrophs associated with the Calthion sites and the high water content or peat composition at these sites^36^. Strong associations between the bacterial OTUs assigned to the Nitrospirales order and the plant species *Juncus effusus* were also discovered at the sites with Calthion alliance. This is a well-studied plant that is frequently deployed for wastewater treatment in artificial wetland systems, notably to remove ammonium compounds from the wastewater^41,42^. The Nitrospirales group includes members that have been reported to be involved in the nitrification step of the nitrogen cycle^43^, which suggests the importance of this plant-bacterial association in the biogeochemical cycles of this mountain microhabitat.

In the sites with Nardion alliance, the bacterial OTUs predominantly co-varied with the plant species *Nardus stricta*. Both the indicator species approach and the correlation network analysis detected a significant enrichment of a little known bacteria family of the Thermogemmatisporaceae within the phylum Chloroflexi and class Ktedonobacteria. The ecological importance of this bacteria family in the grasslands dominated by the Nardion alliance is unknown. The few known Ktedonobacteria have been shown to prevail in such places as oligotrophic^44^ and thermophilic^45^ environments, and have been associated with carbon monoxide oxidation^46^. The family has recently been discovered further in the rhizosphere of *Andropogon gerardii* (big bluestem) grasses^19^, and is worthwhile investigating further.

We further examined whether or not environmental factors had stronger, or equally strong, correlations with the bacterial OTUs and plant species than the correlations between organismal partners. The bacterial OTUs identified in the generalists cluster (Figs 5A and 6A) were highly correlated with soil pH, with Spearman coefficients as high as −0.9193 and 0.9517. This indicates that soil pH is a key driver for many, but not all, of the bacteria in this cluster. Similarly, the generalist plant species were fairly negatively correlated with soil pH, but also with elevation (Fig. 6A,B). This can be explained by the fact that, in this limestone-dominated mountain area, higher elevation ranges tend to have bedrock exposed at the surface, leading to an on average higher soil pH. However, taken across the whole study area, soil pH is weakly correlated with elevation^36^. Other studies also show that plant species can be strongly influenced by both elevation and topography-related factors and soil pH^30,40^. Similarly to the plant-bacterial OTU correlations detected here, King and coworkers^32^ had also shown that across the high-alpine subnival zone in the Colorado Rockies, as much as 31% of the soil bacterial OTUs correlated with various aboveground plant species. Furthermore, Zinger and coworkers^47^ found that plant and bacterial community beta-diversity in the Galibier mountain region in France were positively correlated. Similar positive correlations between bacterial and plant community diversity were also detected in a variety of grasslands^48^ and on different continents^25^. Therefore, as indicated previously by King *et al*.^32^, our study warrants that observed plant-bacteria species correlations at the landscape scale partially result from underlying environmental factors.

Our observations demonstrate that the plant and bacterial communities are distinct across different phytosociological alliances, and that within individual alliances, various environmental drivers are strongly influencing both the plant and bacterial species that are thriving in these habitats. Across multiple alliances, it is evident that soil pH is a key driver of the generalist bacteria and plants (Fig. 6A). We acknowledge that soil pH is correlated to a number of other environmental factors, such as CaO and calcite content, mineral carbon and bulk carbon content, as pointed out earlier^36^. Within the individual alliances, however, alliance-specific environmental factors are important in shaping the respective communities (Figs 6D and S7), on top of which plant-bacteria interactions play a role. Interestingly, even after factoring out the effect of soil pH from the NTI values (Fig. 3B), the vegetation alliances from the neutral pH sites had significantly more phylogenetically clustered bacterial communities compared to alliances from less neutral soils. This suggests that soil neutrality is particularly important for bacterial prosperity, which promotes either directly (through pH selection)^49^ or indirectly (as a result of higher species diversity)^50^ greater bacterial species diversification, relative to less neutral soil environments.

A proportion of the bacterial OTUs associated to the cluster formed by generalist plants (e.g., Fig. 5A) was also correlated with elevation. Given that the relative abundance and richness of individual bacterial OTUs do not typically follow the elevation gradient in the same way as plants do^36,51^, this observed trend is suggestive of plant-driven changes in the abundance of these bacteria. Similar statements could be made for the soil bacteria that were strongly associated with the *Cynosurus cristatus*, *Dactylis glomerata*, *Festuca pratensis*, and *Rananculus acris* plant species. Furthermore, a disproportionately large number of negative correlations was detected between generalist plants and bacterial OTUs, which might be due to the fact that bacteria tend to prefer relatively less acidic conditions^31,36^, while these generalist plant species are relatively more abundant in acidic soils.

In conclusion, we were able to show that bacterial and plant communities (as categorized by the vegetation alliances) co-pattern strongly across the highly heterogeneous mountain grasslands in the western Swiss Alps. This supports our initial hypothesis that there should be some form of equilibrium between plant diversity and soil microbes at most sites, outside the direct plant rhizosphere (which was removed in our analysis). Certain alliances thrive at more distinct environmental conditions than others and different alliances were characterized by the importance of either specialist or generalist plant species and bacterial OTUs, with different survival strategies. Interestingly, also grassland soil fungal communities show marked co-occurrence patterns with plant vegetation alliances^34^. Our results thus point to potentially favorable plant-bacteria interaction patterns at regional scale, which may help to better understand the underlying ecological and evolutionary forces driving these associations.

## Materials and Methods

The study area consists of a ca. 700 km^2^ region in the western Swiss Alps in the canton of Vaud. The sampling area covered an elevation gradient of 800–3,000 m and has been studied for the past 10 years in terms of plant and animal diversity, as well as soil properties and climatic factors (http://rechalp.unil.ch/).

The 100 grassland sites used for the present study were selected according to a random-stratified design along elevation, slope and aspect, and were sampled during summer 2012 (previously described in ref.^36^). A total of 56 edaphic factors was determined for each of the sampling sites, and is described previously^36,40^ (Fig. 2A). Bacterial community structure data were derived from high-throughput sequencing of amplified V5 hypervariable regions of the 16 S rRNA gene and have been described previously^36^. In short, total DNA was isolated from fresh homogenized samples, pooled from five subsamples in a 2- by 2-m plot for every site encompassing the 1–5 cm topsoil (excluding plant roots). DNA was purified using the PowerSoil DNA isolation kit (MO BIO Laboratories, Carlsbad, CA, USA), as further detailed previously^36^. The V5 hypervariable region of the 16 S rRNA gene was amplified from the purified soil DNAs, and paired-end sequenced on an Illumina HiSeq. 2500 platform at the Lausanne Genomic Technologies Facilities^36^. We chose to target the V5 region, because it can be completely covered by paired-end Illumina HiSeq reads (2 × 100 nt)^36^. The sequenced paired-end reads were demultiplexed and quality filtered using in-house scripts, and assembled using PandaSeq^52^. Filtered reads were clustered into OTUs at the 97% similarity threshold using QIIME v.1.7.0^53^ and the gg_13_8 database from Greengenes as a reference^54^. The OTUs per sample were then normalized by rarefaction to the lowest sequence number per site across all sites (99,618 sequences). All sample reads are available from the NCBI Sequence Read Archive under BioProject numbers PRJNA327018 and PRJNA327017.

QIIME was further used to generate the bacterial OTU richness values, weighted and unweighted UniFrac matrices^55^, and the nearest taxon index (NTI^56^). The NTI was calculated for the bacterial community at each site in order to test whether or not the degree of phylogenetic clustering or overdispersion was similar within respective vegetation alliances, and whether or not there were noticeable differences in the phylogenetic composition across alliances that can be explained by the local abiotic factors and/or evolutionary history of the respective alliances. Given the very large difference in community composition at the water-logged Calthion alliance sites in comparison to all of the other grassland habitats, the communities belonging to the Calthion were omitted when calculating the quadratic regression across the NTI values as a function of soil pH, and during further hypothesis testing of groups that became evident after examining the residuals from the quadratic fit.

The plant species richness and cover-abundance data across the area had been collected and compiled between the years 2002 and 2010^30^. The vegetation alliance information for all 100 sites was determined at the time of plant sampling^30^, and is summarized in Table 1.

Co-variation of plant vegetation alliances and bacterial communities (i.e., the retained normalized OTUs per site) was tested in two ways, using multivariate ordinations (general co-variation) and correlation network analysis (individual co-variation). We did not use null model co-occurrence analyses^57,58^, as these are normally used within a same group and cannot handle very large species numbers. We first looked at general patterns of co-variation by grouping the bacterial communities according to the site-specific alliances across a non-metric multidimensional scaling (NMDS) ordination space defined with the bacterial data, calculated from the weighted UniFrac base matrix, using the R package *vegan* (http://www.r-project.org)^59^. In these analyses, the vegetation alliance information was added *a posteriori* in form of color code, but was not included in the calculation itself. Co-inertia was then analyzed in the R package *ade4*^60^ using the NMDS scores of bacterial communities and plant communities, calculated from the weighted UniFrac and Bray Curtis distance matrices, respectively, that were exported from the R package *vegan* to show whether particular groups of plant communities co-occurred with particular groups of soil bacterial communities across environmental gradients. In order to show that the vegetation alliances were significantly clustered within the bacterial NMDS plot, a one-sample *t*-test was used on the ordihull areas (drawn convex hulls connecting the vertices of the points made by the communities on the NMDS plot – using the *ordihull* function) to show that the area occupied by the respective vegetation alliances was significantly smaller than the total area of the points within the bacterial community ordination space. The R package *labdsv* was used to calculate for each vegetation alliance indicator taxa for both bacterial OTUs and plant species, using a *P*-value threshold of 0.05^61^. Plant indicator species were defined according to ref.^61^. Plant species cover abundance across vegetation alliances was represented as a heat-map using the R package *gplots*. Quadratic regression analyses were calculated using the *lm* function in the R base package.

Individual plant species and bacterial OTU co-occurrences were then examined as follows. First, to ensure sufficient statistical power, we restricted our analyses to bacterial OTUs having at least total relative abundances across all sites of more than 100, and to plant species found in at least three sites. We chose a relatively low and different threshold for plant species, in comparison with bacteria, because of their overall lower prevalence across plots and the use of semi-quantitative abundance-dominance instead of absolute abundance information for them. Spearman correlations between these OTUs and plant species groups were then generated by using the *Hmisc* R package. *P*-values from Spearman correlation coefficients were corrected for false-discovery rates (FDR) according to ref.^62^, and only coefficients ≥0.6 or ≤−0.6, and FDR-corrected *P* < 0.05 were kept. Plant-bacterial OTU correlations were visualized in networks produced by the Fruchterman Reingold algorithm^63^ in Gephi v.0.8.2^64^. Striking clusters were named according to abundant plant names, or vegetation alliances, if recognizable. A couple of very small plant-bacterial OTU clusters were excluded from further analysis where neither specific habitats nor specific bacterial OTU indicators could be identified. All clusters are documented in Dataset 1.

In order to determine the importance of underlying environmental factors driving the organism abundances across the landscape, further Spearman and partial Spearman correlations were calculated between the detected co-occurring plant species and bacterial OTUs, and the following nine environmental factors: soil pH, elevation, bulk soil water and total organic carbon content, hydrogen index, δ^13^C, mineral carbon, total N and soluble P. These nine variables had previously displayed a significant impact in shaping the bacterial communities for the study area^36^. The partial correlation coefficients were calculated using the *ppcor* package in R.

## Electronic supplementary material

Supplementary figures

Dataset 1
