# Supplementary material for: Meta-scale mountain grassland observatories uncover commonalities as well as specific interactions among plant and non-rhizosphere soil bacterial communities
Source: Sci Rep. 2018 Apr 10;8:5758. doi: 10.1038/s41598-018-24253-x (PMC5893626; doi:10.1038/s41598-018-24253-x)
Supplement: Supplementary file 1 — Supplementary figures [file 41598_2018_24253_MOESM1_ESM.pdf]

Supplementary Information to:

Meta-scale mountain grassland observatories uncover commonalities as well as specific interactions among plant and non-rhizosphere soil bacterial communities

Erika Yashiro[1], Eric Pinto-Figueroa[2], Aline Buri[3], Jorge E. Spangenberg[3], Thierry Adatte[4], Helene Niculita-Hirzel[5], Antoine Guisan[2,3*], and Jan Roelof van der Meer[1*]

* Shared last authorship

1) Department of Fundamental Microbiology, University of Lausanne, 1015 Lausanne, Switzerland

2) Department of Ecology and Evolution, University of Lausanne, 1015 Lausanne, Switzerland

3) Institute of Earth Surface Dynamics, University of Lausanne, 1015 Lausanne, Switzerland

4) Institute of Earth Sciences, University of Lausanne, 1015 Lausanne, Switzerland

5) Institute for Work and Health, University of Lausanne and Geneva, 1066 Epalinges-Lausanne, Switzerland

Figures S1-S7
Table S1

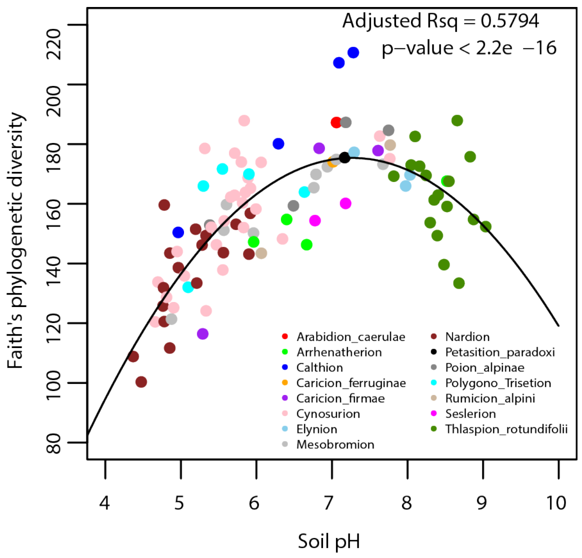

Figure S1. Faith's phylogenetic diversity of the bacterial communities across the soil pH gradient. The communities are color-coded according to the vegetation alliance of the respective sites. The lm function was used to generate a quadratic equation to fit the data points, and the adjusted R squared and p values of the model fit are indicated.

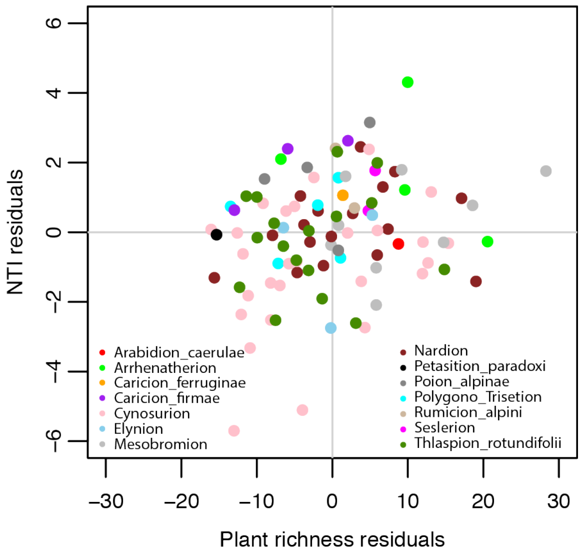

Figure S2. Scatterplot of the bacterial NTI and plant species richness residuals after removing the effect of soil pH. The points are color-coded according to the vegetation alliance to which each site belongs.

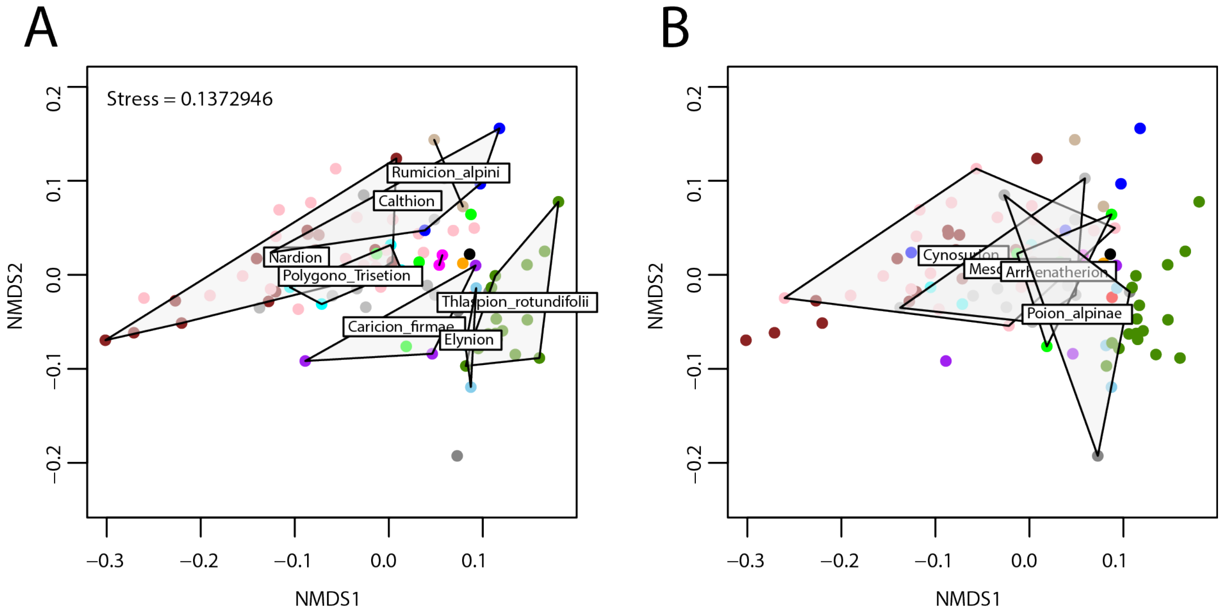

Figure S3. Non-metric multidimensional scaling plots of the bacterial weighted UniFrac matrices. The ordihulls delimit the sites belonging to the respective vegetation alliances. (A) The sites where bacterial indicator OTUs were identified, and (B) the sites where no indicator OTUs were identified.

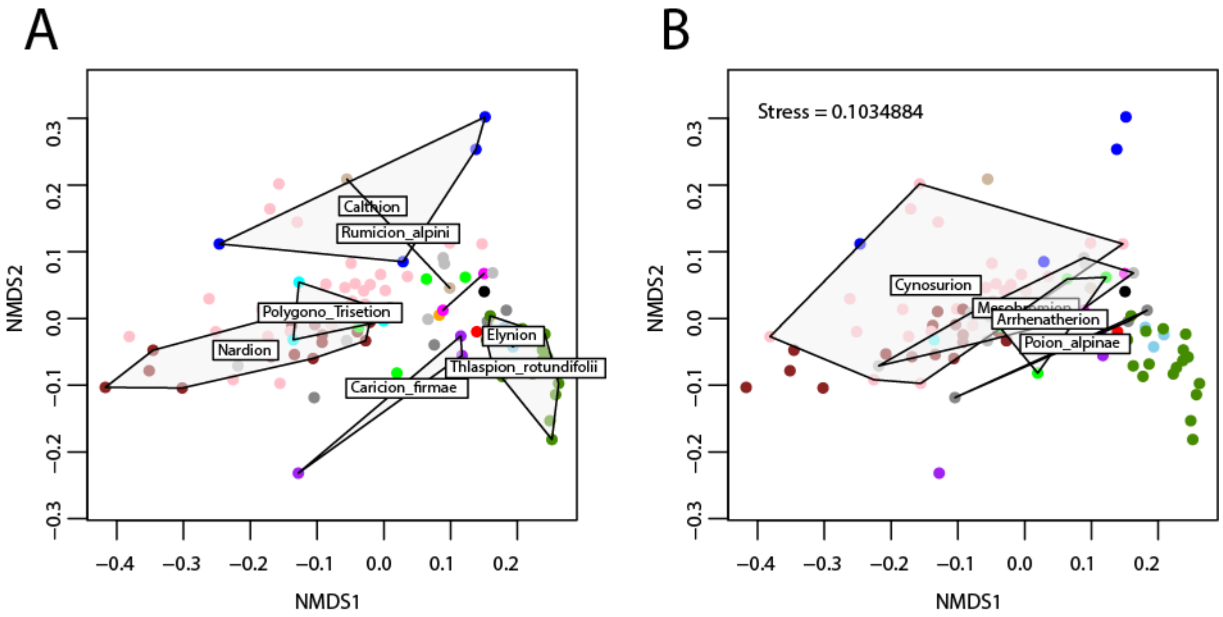

Figure S4. Non-metric multidimensional scaling plots of the bacterial unweighted UniFrac matrices. The ordihulls delimit the sites belonging to the respective vegetation alliances. (A) The sites where bacterial indicator OTUs were identified, and (B) the sites where no indicator OTUs were identified.

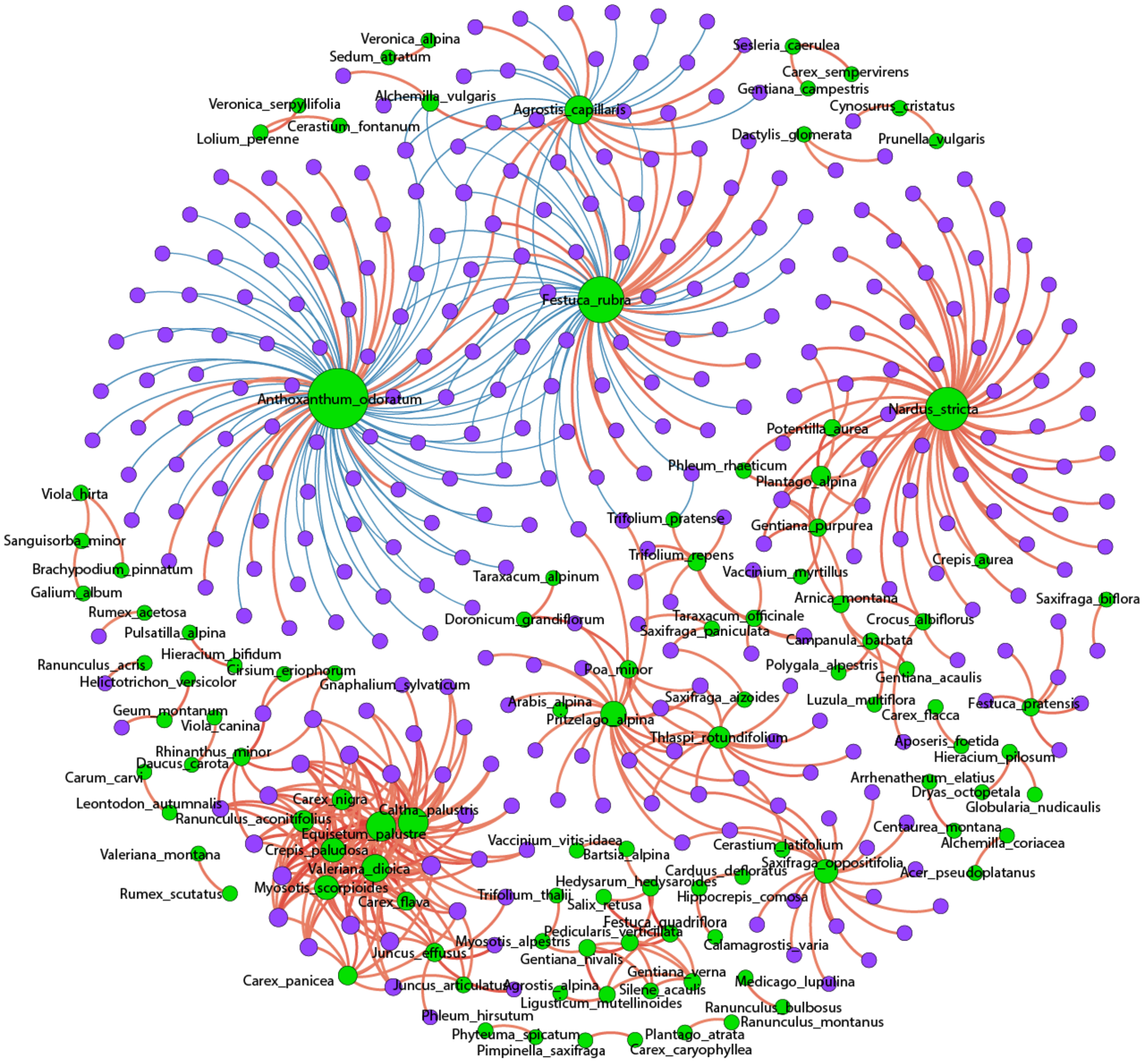

Figure S5. Co-occurrence network from Figure 5, showing the taxonomic identity of the plant nodes.

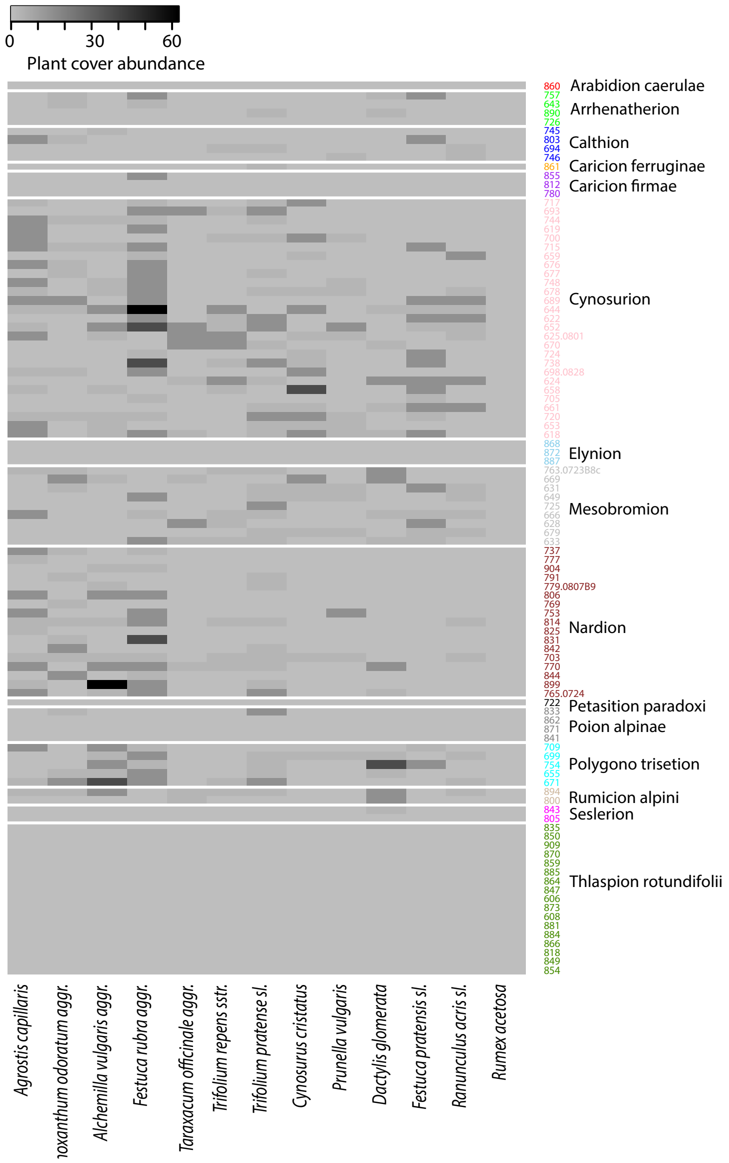

Figure S6. Heatmap of the prevalence of plant species across the vegetation alliances. Only those bacterial-associated plant species belonging to co-occurrence clusters (Figure 3) without indicator OTU assignments are shown.

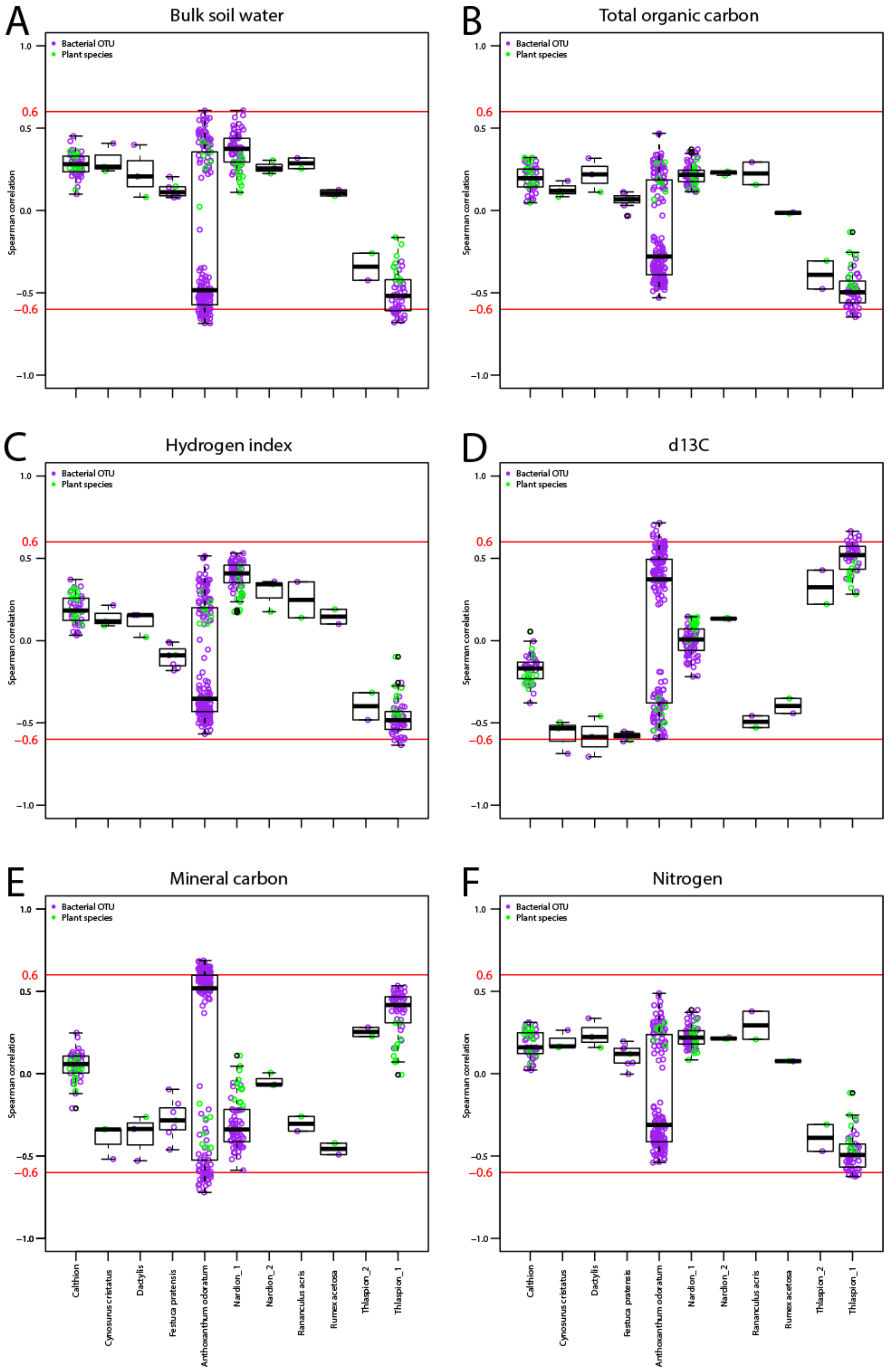
A
Bulk soil water
B
Total organic carbon
C
Hydrogen index
D
d13C
E
Mineral carbon
F
Nitrogen
Bacterial OTU
Plant species
Spearman correlation
1.0
0.6
0.5
0.0
−0.5
−0.6
−1.0
Calthion
Cynosurus cristatus
Dactylis
Festuca pratensis
Anthoxanthum odoratum
Nardion_1
Nardion_2
Ranunculus acris
Rumex acetosa
Thlaspion_2
Thlaspion_1

Figure S7. Effects on different environmental variables on Spearman correlation coefficients within the respective co-occurrence clusters harboring both plant and bacterial nodes. The correlation analyses were done between the plant species (green) and bacterial OTUs (purple) and (A) soil bulk soil water content, (B) total organic carbon content, (C) soil hydrogen index, (D) stable isotopic carbon ratio, (E) soil mineral carbon content, and (F) total nitrogen content. The red line indicates Spearman coefficients 0.6 and -0.6, which were the coefficient thresholds used to build the plant-bacteria co-occurrence networks in Figure 5. The purple and green circles indicate individual bacterial OTUs and plant species, respectively.

Table S1. List of plant indicator species of those alliances represented by more than one site in the study.

**Plant species**	**Alliance**
Fragaria vesca	Arrhenatherion
Caltha palustris	Calthion
Equisetum palustre	Calthion
Ranunculus aconitifolius	Calthion
Valeriana dioica	Calthion
Juncus articulatus	Calthion
Juncus filiformis	Calthion
Saxifraga paniculata	Elynion
Sanguisorba minor sl.	Mesobromion
Plantago media	Medobromion
Potentilla aurea	Nardion
Myosotis alpestris	Poion alpinae
Trifolium thalii	Poion alpinae
Geranium sylvaticum	Polygono trisetion
Tussilago farfara	Rumicion alpini
Calamagrostis varia	Seslerion
Scabiosa lucida	Seslerion
Hippocrepis comosa	Seslerion
Pritzelago alpina sstr.	Thlaspion rotundifolii
Poa minor	Thlaspion rotundifolii
